# Salt glands in exo‐recretohalophytes: Development, physiological functions, and prospects for improving crop salt tolerance

**DOI:** 10.1111/jipb.70141

**Published:** 2026-01-19

**Authors:** Limin Wang, Junyan Xie, Yiping Zou, Chunliang Yao, Hai Fan, Chenqi Shen, Wenyan Zhou, Jingran Qin, Xinke Zhang, Baoshan Wang, Jian Zhang, Guoliang Han

**Affiliations:** ^1^ Shandong Provincial Key Laboratory of Stress Plant Biology and Genetic Improvement, College of Life Sciences Shandong Normal University Jinan 250014 China; ^2^ National Center of Technology Innovation for Comprehensive Utilization of Salin‐Alkali Land Agricultural High‐tech Industrial Demonstration Area of the Yellow River Delta of Shandong Province Dongying 257000 China; ^3^ Dongying Institute, Shandong Normal University Dongying 257000 China

**Keywords:** exo‐recretohalophyte, regulatory network, salt gland, salt secretion, salt stress

## Abstract

In exo‐recretohalophytes, specialized structures known as salt glands secrete excess salt ions from plant tissues, thereby maintaining intracellular ion homeostasis and sustaining normal metabolism under salt stress. Based on their cellular composition, salt glands can be unicellular, bicellular, or multicellular, and they originate from undifferentiated precursor cells known as multipotent epidermal stem cells. A complex regulatory network drives the division and differentiation of these cells into functional salt‐secreting structures. Three hypotheses have been proposed to explain the physiological mechanisms underlying salt secretion by salt glands, each supported by experimental evidence: The osmotic mechanism, the reverse pinocytosis mechanism, and the animal‐like fluid transport mechanism. This review summarizes the structural characteristics, developmental processes, salt secretion mechanisms, and potential applications of salt glands in exo‐recretohalophytes, providing a foundation for future studies on salt gland biology and their utilization in developing salt‐tolerant crops.

## Graphical abstract

This review examines salt glands in exo‐recretohalophytes, in which epidermal stem cells differentiate into unicellular, bicellular, or multicellular salt glands. Salt ions are transported to the leaves via the transpiration stream and enter salt glands through symplastic and apoplastic pathways. Finally, salt glands actively secrete salt ions from the plant.

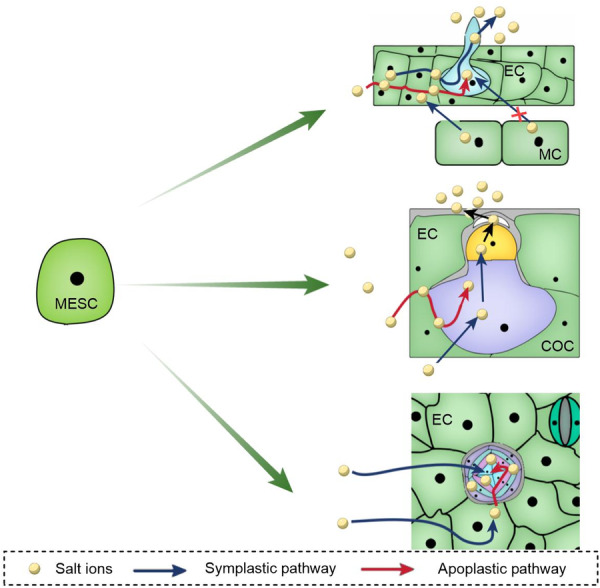

## INTRODUCTION

The distribution and continual expansion of saline‐alkaline soils have become a global ecological and agronomic issue (FAO, 2024; Wang et al., 2024b; Yuan et al., 2025), severely limiting the arable land available for crop production and directly threatening food security. Coastal saline‐alkaline soils have selected for plants with unique salinity tolerance mechanisms that enable them to complete their life cycles in high‐salt environments; these salt‐tolerant plants are known as halophytes (Meng et al., 2018). In contrast, non‐halophyte plant species, including most major food crops, have generally evolved in low‐salinity environments, lack specialized salinity tolerance mechanisms, and can only complete their life cycles under low‐salt conditions (Munns and Tester, 2008).

Non‐halophytes possess several mechanisms to cope with the osmotic component of salt stress. For example, they maintain water uptake by cells and cellular turgor pressure by accumulating compatible osmolytes such as proline and betaine (Mahmood et al., 2020; Li et al., 2022; Liang et al., 2023). To mitigate the ionic toxicity associated with high salinity, such as the damage caused by the accumulation of sodium (Na^+^) and chloride (Cl^−^) ions, non‐halophytes use three principal strategies: sequestration of ions into vacuoles, reduction of ion transport from roots to shoots, and extrusion of the ions out of cells (Teakle and Tyerman, 2010; Yang and Guo, 2018; Wang et al., 2022c). To resist oxidative damage associated with salt stress, non‐halophytes activate a reactive oxygen species (ROS)‐scavenging system comprising both enzymatic and non‐enzymatic antioxidants (Das and Roychoudhury, 2014; Nadarajah, 2020; Zhang et al., 2023b). These mechanisms generally only enable non‐halophytes to complete their life cycle under low‐salinity conditions, but are insufficient to support their survival under sustained high‐salinity stress.

In addition to the above adaptations, halophytes have evolved specialized mechanisms that enable them to tolerate high‐salinity environments. Halophytes are classified as euhalophytes, pseudohalophytes, and recretohalophytes, based on their primary salinity tolerance strategies (Zelm et al., 2020; He et al., 2026). Euhalophytes, such as *Suaeda salsa* (L.) Pall., have fleshy leaves and thin‐walled tissues that allow localized storage of salt ions within their succulent organs and cellular vacuoles (Qiang et al., 2022; Cheema et al., 2025). Pseudohalophytes, such as *Phragmites australis* (Cav.) Trin. ex Steudel, restrict salt uptake via specialized barriers on their root surfaces (Gonasageran, 2021; Liu et al., 2023a). Recretohalophytes, in contrast, secrete or temporarily store excess salt ions using specialized salt‐secreting structures. Recretohalophytes can be further divided into exo‐recretohalophytes and endo‐recretohalophytes (Breckle, 1990; Wu et al., 2020). Exo‐recretohalophytes, such as *Limonium bicolor* (Bunge) Kuntze, secrete salt ions via salt glands located on their leaf surface (Yuan et al., 2015b; Wang et al., 2022a). Endo‐recretohalophytes, such as the *Mesembryanthemum crystallinum* L., temporarily store excess salt ions in salt bladders; upon rupture of these bladders, the salt ions are released from the plant (Barkla et al., 2012; Hao et al., 2013). Exo‐recretohalophytes have been more extensively studied than endo‐recretohalophytes to date, and the structure and function of their salt glands remain topics of particular interest.

In this review, we synthesize current knowledge on salt glands, with a focus on their structural characteristics, development, and salt secretion mechanisms, as well as the prospects for applying the principles of salt glands to non‐halophytes. A comprehensive analysis of these structures will help to refine the theoretical framework of salinity tolerance in exo‐recretohalophytes and provide essential theoretical and technical guidance for future research on the development and utilization of exo‐recretohalophyte resources.

## SALT GLAND STRUCTURES

Currently, approximately 370 recretohalophyte species have been identified across 96 genera and 14 families, including 161 exo‐recretohalophytes with salt glands (Yuan and Wang, 2021). These salt glands are mainly distributed on the upper and lower leaf epidermis, with some species also possessing specialized glands at the petiole base (Céccoli et al., 2015; Leng et al., 2018; Zhao et al., 2023). Additionally, significant structural differences in salt glands exist between plant groups: Monocots mainly possess unicellular or bicellular salt glands, whereas dicots mainly possess multicellular salt glands (Dassanayake and Larkin, 2017).

### Structure of unicellular salt glands

**Figure 1:**
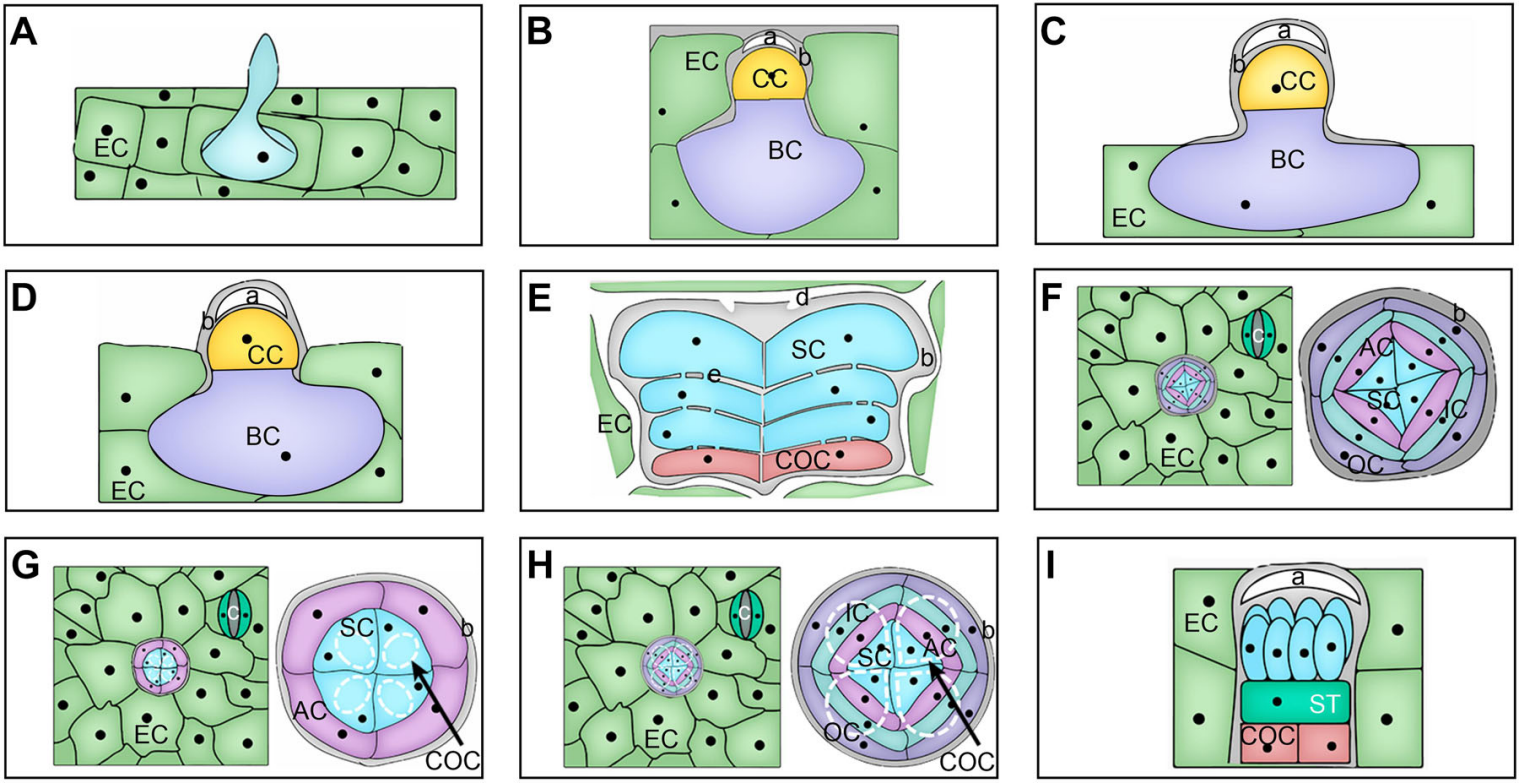
Schematic diagram of salt gland structures Unicellular salt gland: **(A)** *Oryza coarctata*. Bicellular salt glands: **(B)** *Distichlis spicata, Zoysia sinica*, and *Sporobolus anglicus*, **(C)** *Bouteloua gracilis* and *Buchloe dactyloides*, and **(D)** *Dinebra retroflexa* and *Dactyloctenium aegyptium*. Multicellular salt gland structure: **(E)** *Tamarix chinensis*, **(F)** *Limonium bicolor*, **(G)** *Limonium aureum*, **(H)** *Limonium sinense*, and **(I)** *Avicennia marina*. Abbreviations: a, collecting cavity; b, cuticle; c, stomata; d, secretory pore; e, plasmodesmata; AC, accessory cell; BC, basal cell; CC, cap cell; COC, collecting cell; EC, epidermal cell; IC, inner cup cell; OC, outer cup cell; SC, secretory cell; ST, stalk cell.

Limited research has been conducted on unicellular salt glands that are typically found in wild relatives of rice (*Oryza sativa* L.), such as *Oryza coarctata* (Roxb.) (Garg et al., 2014; Litalien and Zeeb, 2020). The salt glands of *O. coarctata* are single specialized secretory cells (SCs) (Figure 1A) that originate from the epidermis and are considered modified trichomes (Rajakani et al., 2019). These salt glands have an electron‐dense vacuole and a thickened cell wall (Bal and Dutt, 1986; Flowers et al., 1990; Litalien and Zeeb, 2020). Multiple plasmodesmata connect each salt gland to adjacent epidermal cells, and only the tip of the salt gland is visible above the leaf surface (Koteyeva et al., 2022).

### Structure of bicellular salt glands

In general, bicellular salt glands consist of a large basal cell (BC) and a smaller cap cell (CC) (Figure 1B–D). These two cells are connected by plasmodesmata and function cooperatively to accumulate, concentrate, and secrete salt taken up by the roots (Dassanayake and Larkin, 2017). Bicellular salt glands are classified into three types based on their position relative to the epidermis and the strength of their secretory function (Semenova et al., 2010). The first type is sunken into the epidermis and shows strong secretory activity, as observed in *Distichlis spicata* (L.) Greene, *Zoysia sinica* Hance, and *Sporobolus anglicus* (C.E. Hubb.) P.M. Peterson & Saarela (Figure 1B) (Lu, 2006; Semenova et al., 2010; Koteyeva et al., 2022). The second type protrudes above the epidermis and shows weak secretory activity; examples include *Bouteloua gracilis* (H. B. K.) Lag. ex Steud. and *Buchloe dactyloides* (Nutt.) Engelm. (Figure 1C) (Liphschitz and Waisel, 1982; Zhang et al., 2013). The third type is intermediate in both morphology and function, as observed in *Dinebra retroflexa* (Vahl) Panz. and *Dactyloctenium aegyptium* (L.) Willd. (Figure 1D) (Yuan et al., 2015a; Ma and Yuan, 2023).

### Structure of multicellular salt glands

Research on the structure of multicellular salt glands has primarily focused on dicots, particularly the *Tamarix* and *Limonium* genera (Thomson and Platt–Aloia, 1985; Wei et al., 2020; Meng et al., 2024). Multicellular salt glands vary in size and distribution, but are commonly spherical or disc‐shaped, and the SCs are completely surrounded by the cuticle. Generally, multicellular salt glands consist of two main components: A basal group of collecting cells (COCs) and a distal group of SCs, which are typically embedded within the epidermal cell layer (Dassanayake and Larkin, 2017).

A detailed study conducted in the 1960s described the salt glands of *Tamarix chinensis* Lour., revealing that each gland comprises eight cells (Thomson and Liu, 1967; Shimony and Fahn, 1968; Wei et al., 2020). The inner layer consists of two COCs characterized by large vacuoles, and the outer layer contains six SCs with dense cytoplasm (Figure 1E). Numerous plasmodesmata are present between the SCs and COCs, and the SCs are rich in mitochondria and possess cell wall protrusions. The entire salt gland is enclosed by the cuticle (Thomson and Liu, 1967; Bosabalidis, 2010; Chen et al., 2023). These structural features contribute to the strong secretion capacity of salt glands in *T. chinensis*.

Similarly, research on *Limonium* dates back to the 1960s (Ziegler and Lüttge, 1967). In *L. bicolor*, each salt gland is composed of sixteen cells (Figure 1F): Four central SCs, each surrounded by four accessory cells (ACs). Externally, these ACs are enclosed by four inner cup cells (ICs), which in turn are surrounded by four outer cup cells (OCs) (Yang et al., 2024). Salt gland cells possess larger nuclei than those of typical mesophyll or epidermal cells and contain abundant mitochondria, Golgi apparatus, and small vesicles, but lack large central vacuoles and chloroplasts. Numerous plasmodesmata connect salt gland cells to the surrounding mesophyll cells. Additionally, the outer surface of the salt gland is covered by the cuticle (Yuan et al., 2015b). Four secretion pores have been identified on the outer surface, providing channels for the efflux of salt ions (Feng et al., 2014).

In addition to *L. bicolor*, salt glands have been identified in other *Limonium* species, including *Limonium aureum* (L.) Hill., *Limonium sinuatum* (L.) Mill., *Limonium sinense* (Girard) Kuntze, *Limonium franchetii* (Debeaux) Kuntze, *Limonium gmelinii* (Willd.) Kuntze, *Limonium otolepis* (Schrenk) Kuntze, *Limonium myrianthum* (Schrenk) Kuntze, and *Limonium chrysocomum* (Kar. & Kir.) Kuntze. Among these, the salt gland of *L. sinuatum* closely resembles that of *L. bicolor* in morphology and cellular composition (Mi et al., 2021). By contrast, the salt gland of *L. aureum* consists of twelve cells: Four SCs, four ACs surrounding the SCs, and four COCs located deep within the gland (Figure 1G) (Ni et al., 2012; Mi et al., 2021). Furthermore, the salt glands of *L. sinense*, *L. franchetii*, *L. gmelinii*, *L. otolepis*, *L. myrianthum*, and *L. chrysocomum* contain twenty cells: Four SCs, four ACs arranged in a circular pattern, four ICs, four OCs, and four COCs at the base (Figure 1H) (Zhou et al., 2006; Xing et al., 2012; Mi et al., 2021).

Mangroves are widely distributed in the intertidal zone of tropical and subtropical coasts, and some species possess typical multicellular salt glands. The *Avicennia marina* (Forsk.) Vierh. has been relatively well studied in this regard (Chen et al., 2010; Tan et al., 2013; Wei et al., 2022). Its salt gland is composed of eleven cells: Two COCs, one stalk cell (ST), and eight SCs (Figure 1I). Salt gland cells contain abundant plasmodesmata and mitochondria, with plasmodesmata mediating intercellular material exchange and mitochondria providing energy for ion transport (Balsamo and Thomson, 1993; Wei et al., 2022).

The unique structural features of those salt glands underlie their high salt secretion rates.

## SALT GLAND DEVELOPMENT: MORPHOGENESIS AND MOLECULAR REGULATION

The three aforementioned types of salt glands share a common origin, all deriving from multipotent epidermal stem cells (MESCs). However, research on unicellular and bicellular salt glands remains limited. Therefore, the discussion of the regulatory networks governing salt gland formation will focus primarily on the multicellular salt glands of *L. bicolor*. In the development of salt glands in *L. bicolor*, the division and differentiation of MESCs proceed in a highly ordered manner under strict regulation, orchestrated by a complex network comprising transcription factors, other regulatory proteins, and long non‐coding RNAs (lncRNAs). In addition to these intrinsic regulatory networks, several exogenous factors influence salt gland development.

### Salt glands originate from MESCs

**Figure 2:**
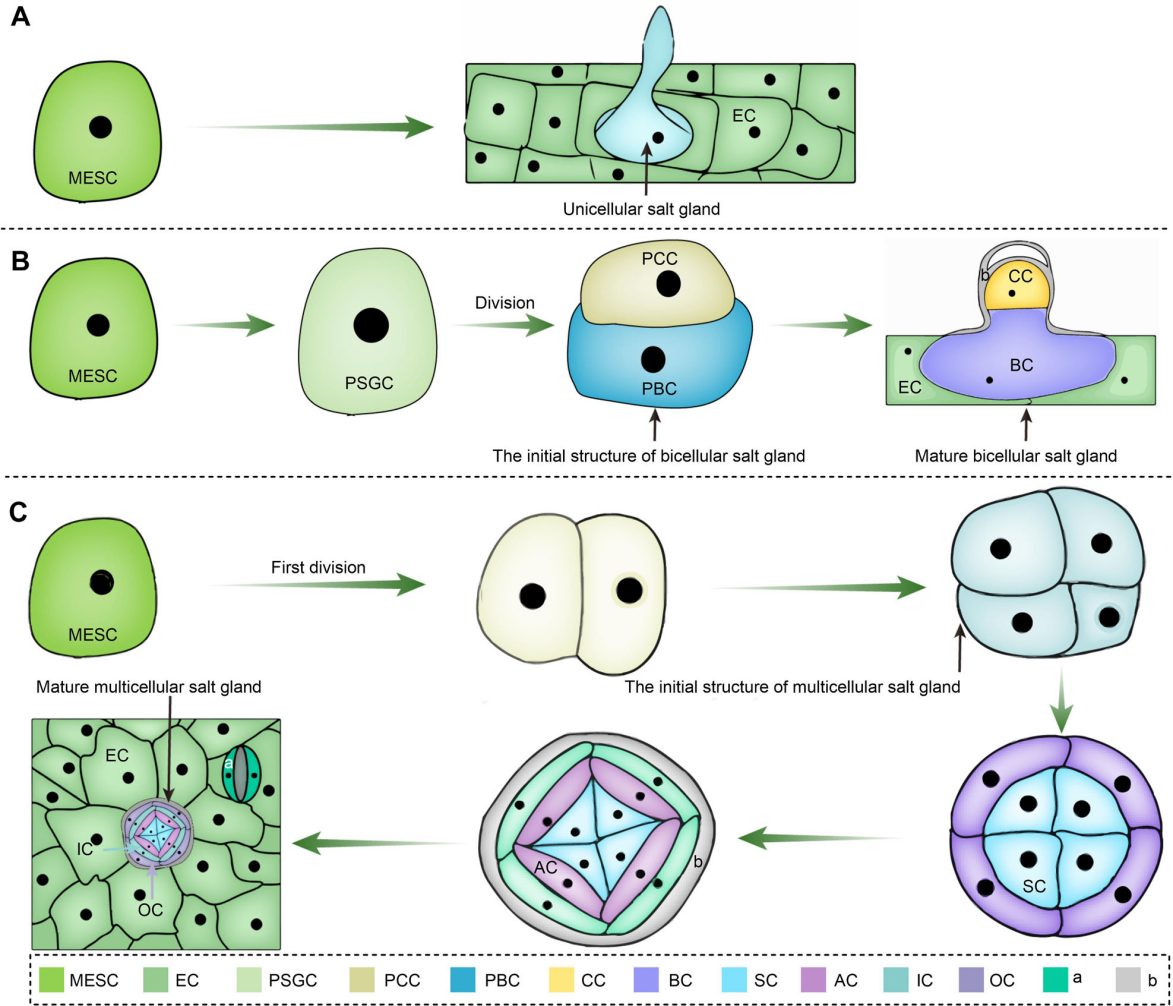
Salt gland development process Schematic models depict the development of **(A)** unicellular (*Oryza coarctata*), **(B)** bicellular (*Zoysia sinica*), and **(C)** multicellular (*Limonium bicolor*) salt glands from MESCs. Abbreviations: a, stomata; b, cuticle; AC, accessory cell; EC, epidermal cell; IC, inner cup cell; MESC, multipotent epidermal stem cell; OC, outer cup cell; PBC, primitive basal cell; PCC, primitive cap cell; PSGC, primitive salt gland cells; SC, secretory cell.

All three types of salt glands originate from MESCs, but they differ markedly in their differentiation and development. Unicellular salt glands, as in *O. coarctata*, develop from rapidly dividing MESCs at the base of new leaves, forming protrusions that are perpendicular to the epidermal cell layer (Figure 2A) (Flowers et al., 1990; Larkin et al., 2003). In bicellular salt glands, as in *Z. sinica*, the cytoplasm of the MESC becomes denser, its nucleus enlarges, and the cell undergoes vertical growth perpendicular to the leaf surface, forming a protruding, primitive salt gland cell (PSGC). Each of these cells then undergoes one round of asymmetric division to form two daughter cells, one of which develops into a primitive cap cell (PCC). The primitive PCC no longer divides and differentiates into a mature CC. The other develops into a primitive basal cell (PBC), which also ceases division and differentiates into a mature BC (Figure 2B) (Lu, 2006). The formation of multicellular salt glands, using *L. bicolor* as an example, involves five rounds of cell division to generate a mature 16‐celled complex (Yuan et al., 2015b). The development of salt glands has been described in detail, based on observations of MESC divisions (Figure 2C). The initial MESC undergoes a first division to form two new MESCs, followed by a second division to generate a four‐celled initial salt gland structure. These two early divisions represent the fate‐determining stages of salt gland formation. During the third division, the four cells divide and differentiate: The two inner cells give rise to four SCs, while the two outer cells produce four salt gland stem cells, forming an eight‐celled structure. In the fourth division, the peripheral salt gland stem cells differentiate into four ACs that surround the inner SCs, while an additional four stem cells form outside the ACs, yielding a 12‐celled intermediate structure. At this stage, the cuticle develops to enclose the salt gland. The fifth and final division involves the four peripheral stem cells, and results in their differentiation into four ICs and four OCs, which surround the ACs. This final arrangement, with SCs, ACs, ICs, and OCs organized radially from the center outward, completes the formation of the mature 16‐celled salt gland (Wiehe and Breckle, 1990; Yuan et al., 2015b).

### Molecular regulation of salt gland development

Current research on unicellular salt glands has been primarily focused on *O. coarctata*. However, progress in this field has been constrained by the species’ narrow coastal distribution, low seed germination rate, and limited availability of genomic resources (Mondal et al., 2018; Zhao et al., 2023; Berqdar et al., 2025). Comparative genomic studies have revealed that a homolog of *TRANSPARENT TESTA GLABRA 1* (*LbTTG1*), a key regulatory gene governing multicellular salt gland development in *L. bicolor*, is not found in *O. coarctata* (Chen et al., 2024), implying that the developmental regulatory networks may differ between the two types of salt glands. Moreover, *OcWox3B*, which shows salt gland‐specific expression in *O. coarctata* (Rajakani et al., 2019), is proposed to be involved in unicellular salt gland development, and yet, direct evidence regarding its developmental process and functional validation remains scarce.

Research on bicellular salt gland development remains limited, primarily due to two major constraints. First, genomic resources have been scarce, with only a few species having had their genomes assembled until recently (Park et al., 2025). Second, the lack of stable genetic transformation systems in relevant grass species has hindered functional genetic studies (Zhang et al., 2005), thereby limiting the elucidation of the underlying molecular mechanisms.

Compared to unicellular and bicellular salt glands, the multicellular salt gland system in *L. bicolor* possesses a more established research system, supported by both a chromosome‐level genome assembly (Yuan et al., 2022) and an established genetic transformation system. These resources have greatly facilitated investigations into its developmental mechanisms. Studies indicate that multiple transcription factor families act in a coordinated manner to regulate salt gland development, including V‐myb avian myeloblastosis viral oncogene homolog (MYB), WRKY, NAM, ATAF, CUC (NAC), APETALA2/ETHYLENE‐RESPONSIVE ELEMENT BINDING FACTOR (AP2/ERF), and basic helix–loop–helix (bHLH) families. Additionally, proteins such as mitogen‐activated protein kinases, zinc finger proteins, WD40‐repeat proteins, and importin‐β proteins also play important roles in salt gland development, along with lncRNAs and proteins of unknown function.

**Figure 3:**
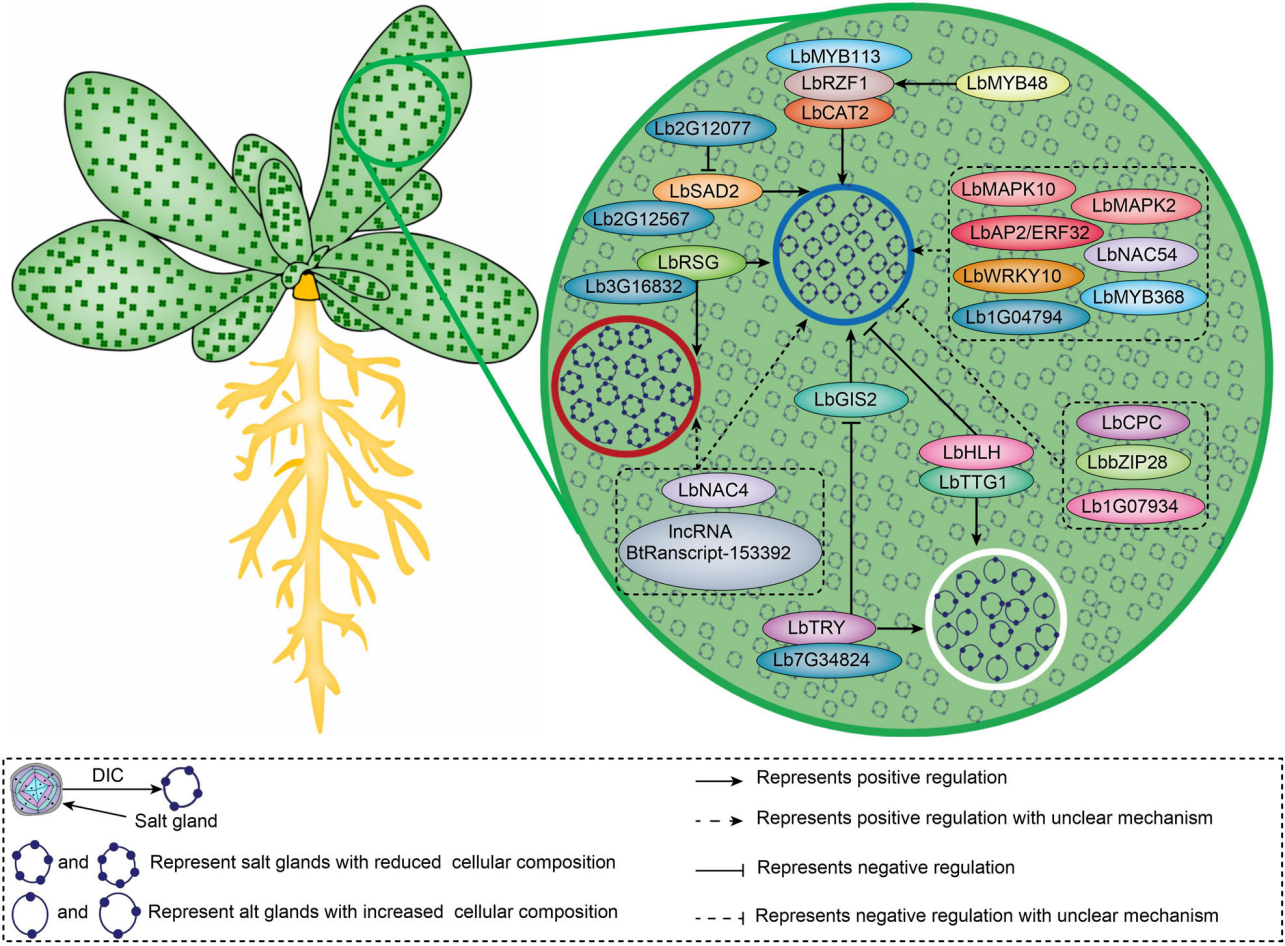
Molecular regulatory mechanisms of salt gland development in *Limonium bicolor* The network of transcription factors, functional proteins, and lncRNAs modulates salt gland density and/or cellular structure. Normal glands (blue circles) display 4 fluorescent foci, increasing to 5–6 with more cells (red circles) or decreasing to 2–3 with fewer cells (white circles).

A subset of the genes and proteins associated with multicellular salt gland development in *L. bicolor* functions as positive regulators, and their roles and interaction networks have been partially elucidated. Under salt stress, the R1‐MYB transcription factor gene *LbMYB48* is strongly induced. Silencing of *LbMYB48* using virus‐induced gene silencing (VIGS) reduces salt gland density on the epidermis, whereas its overexpression significantly increases salt gland density. LbMYB48 positively regulates the expression of its target gene RING ZINC FINGER PROTEIN 1 (*LbRZF1*), which encodes a RING zinc finger protein. LbRZF1 was later shown to interact with LbMYB113 and catalase 2 (LbCAT2), enhancing their stability and thereby positively regulating salt gland density (Han et al., 2022b; Yang et al., 2024) (Figure 3). SUPER SENSITIVE TO ABA AND DROUGHT2 (LbSAD2), an importin‐β, can interact with the protein encoded by *Lb2G12567* to positively regulate the density of salt glands. Conversely, the uncharacterized protein encoded by Lb2G12077 negatively regulates *LbSAD2* expression, resulting in reduced salt gland density (Ma et al., 2025) (Figure 3). In addition, several genes responsive to phytohormones or salt stress have been shown to positively regulate salt gland density, including *GLABROUS INFLORESCENCE STEMS 2* (*LbGIS2*) (Zhao et al., 2024), *Lb1G04794* (Jiao et al., 2022), *MITOGEN‐ACTIVATED PROTEIN KINASE 2* (*LbMAPK2*) and *MITOGEN‐ACTIVATED PROTEIN KINASE 10* (*LbMAPK10*) (Zhang et al., 2023a), *LbWRKY10* (Zhu et al., 2024a), *LbNAC54* (Zhang et al., 2024), *LbAP2/ERF32* (Jiang et al., 2024), and *LbMYB368* (Zhu et al., 2025a) (Figure 3). Some positive regulators of salt gland development influence both the density and the cellular composition of salt glands. A typical sixteen‐cell salt gland in *L. bicolor* displays four fluorescent foci under ultraviolet light, each corresponding to a secretory pore. Overexpression of *REDUCED SALT GLAND* (*LbRSG*), which encodes a plasma membrane‐localized transmembrane protein, not only increased salt gland density but also resulted in abnormal salt glands with five or six fluorescent foci (Figure 3), suggesting an increase in the number of cells per salt gland. Notably, LbRSG interacts with the protein encoded by *Lb3G16832*, and together, they promote salt gland development (Zhou et al., 2024) (Figure 3). Moreover, genes such as *LbNAC4* (Zhao et al., 2023) and the lncRNA *Btranscript‐153392* (Wang et al., 2024d) are highly expressed during salt gland development and positively regulate both cellular composition and salt gland density (Figure 3). Although the functions of these genes have been preliminarily validated, their specific regulatory mechanisms remain elusive and warrant further investigation.

Ongoing research has identified several negative regulators of multicellular salt gland development in *L. bicolor*, including *CAPRICE* (*LbCPC)* (Zou et al., 2023), the basic leucine zipper gene *BASIC LEUCINE ZIPPER 28* (*LbbZIP28*) (Meng et al., 2024), and the bHLH family gene *Lb1G07934* (Wang et al., 2024c) (Figure 3), although their specific regulatory mechanisms have yet to be fully elucidated. Overexpression of *LbTTG1* or *LbHLH*—encoding two interacting proteins—resulted in decreased salt gland density and the formation of abnormal salt glands with only two or three fluorescent foci, whereas knockout of either gene produced the opposite phenotype (Yuan et al., 2022) (Figure 3). Additionally, single‐cell RNA sequencing studies have underscored the importance of cytokinin in salt gland development and identified two genes specifically expressed during early salt gland development: The cytokinin‐related gene *LbGIS2* and the R3‐MYB gene *TRIPTYCHON* (*LbTRY*) (Leng et al., 2021; Zhao et al., 2024). LbTRY interacts with LbGIS2 and the protein encoded by *Lb7G34824*. Silencing of *LbTRY* or *Lb7G34824* increased salt gland density; moreover, *LbTRY*‐silenced plants developed abnormal salt glands with five or more fluorescent foci. In contrast, silencing *LbGIS2* led to decreased salt gland density. These findings suggest that the LbTRY‐Lb7G34824 complex may bind to LbGIS2 and inhibit its function, thereby negatively regulating salt gland development by modulating the cytokinin signaling pathway (Figure 3) (Zhao et al., 2024).

Notably, the following *L. bicolor* genes are homologous to trichome development genes in *Arabidopsis thaliana* (L.) Heynh. and regulate trichome development in this non‐halophyte plant species: *LbTTG1* (Yuan et al., 2022), *LbHLH* (Yuan et al., 2022), *LbCPC* (Zou et al., 2023), *LbTRY* (Zhao et al., 2024), and *LbSAD2* (Ma et al., 2025). Indeed, heterologous expression of any of these *L. bicolor* genes in *Arabidopsis* affects trichome development, indicating that salt glands and trichomes may share conserved core regulatory components. However, the MYB–bHLH–WD40 (MBW) complex, which regulates unicellular trichomes in *Arabidopsis* and is composed of GLABRA (GL1) or MYB23, TTG1, and GL3 or ENHANCER OF GL1 3 (EGL3), cannot form in the salt glands of *L. bicolor* or in the multicellular trichomes of *Solanum lycopersicum* L. and *Nicotiana benthamiana* Domin (Serna and Martin, 2006; Pattanaik et al., 2014; Ying et al., 2020; Yuan et al., 2022). This functional distinction highlights a regulatory divergence between unicellular trichomes and certain multicellular epidermal structures—specifically, salt glands and multicellular trichomes. Furthermore, hydathodes (Lüttge, 2019; Mehltreter et al., 2022) and oil glands (Xie et al., 2024; Wang et al., 2024a) in non‐halophytes also share structural and functional similarities with salt glands. However, whether the developmental mechanisms of these structures and salt glands are conserved remains unknown, representing a crucial direction for future research.

In summary, the development of salt glands is a finely regulated process that integrates the functions of numerous proteins within a complex regulatory network. A comprehensive list of proteins currently known to be involved in this process is provided in Table S1.

### Internal and external factors that influence salt gland development

Salt stress, specific ions (e.g., calcium [Ca^2+^] ions), and phytohormone treatment are key environmental and physiological factors regulating the development of plant salt glands. The response of salt gland development to these factors has been investigated in the model halophyte *L. bicolor*, and we summarize the specific regulatory effects and mechanisms below.

Salt stress directly triggers adaptive modifications in the salt glands of *L. bicolor*, which help balance cellular Na^+^ levels. When plants are irrigated with a 300 mM NaCl solution to simulate a high‐salinity natural habitat, they show significant physiological adaptations. On the one hand, Na^+^ ions accumulate extensively within cells, leading to inhibition of growth; on the other hand, plants actively increase the density of salt glands, which expel excess Na^+^ ions from cells, enhancing the ability of *L. bicolor* to resist salt stress. This response alleviates the toxicity of Na^+^ accumulation in cells to some extent (Zhu et al., 2024b).

To investigate the effects of Ca^2+^ ions on salt gland development and the salt secretion rate, seedlings of *L. bicolor* were treated with 200 mM NaCl supplemented with 0, 5, or 20 mM Ca^2+^ for 15 days. The application of 5 or 20 mM Ca^2+^ resulted in greater leaf area and total number of salt glands compared to treatment with NaCl alone, with 20 mM Ca^2+^ showing a greater effect; in addition, treatment with 5 mM Ca^2+^ or 20 mM Ca^2+^ equally enhanced salt gland density (Ding et al., 2010).

Phytohormones also play regulatory roles in salt gland development in *L. bicolor*. For example, exogenous application of 0.22 μM 6‐benzylaminopurine (6‐BA), 0.03–0.1 mM methyl jasmonate (MeJA), and 5 μM melatonin under normal growth conditions all promoted salt gland density (Yuan et al., 2018; Li et al., 2020; Liu et al., 2023b). In addition, when experiencing salt stress imposed by irrigation with a 300 mM NaCl solution, the application of 10 μM abscisic acid (ABA) increased salt gland density in *L. bicolor* and enhanced its resistance to salt stress (Zhu et al., 2024b).

## SALT SECRETION MECHANISM OF SALT GLANGS

The precise operation of the salt gland development regulatory network lays a critical structural and molecular foundation for its salt‐secreting function. On this basis, the salt‐secreting process of halophytes can be described as follows: Salt is first transported to mesophyll or epidermal cells, then transferred to salt gland cells via intercellular transmission, and finally excreted to the external environment via the active secretion mechanism of salt glands, thereby achieving the removal of toxic ions. It is noteworthy that although the structure of salt glands is a core factor determining their salt‐secreting capacity, the actual salt‐secreting efficiency is not constant but shows multi‐dimensional differences; meanwhile, this salt‐secreting process is also subject to the synergistic regulation of a variety of environmental conditions and phytohormones. In addition to secreting salt ions, salt glands can also secrete other inorganic ions and organic compounds.

### Transport of salt from mesophyll or epidermal cells to salt glands

**Figure 4:**
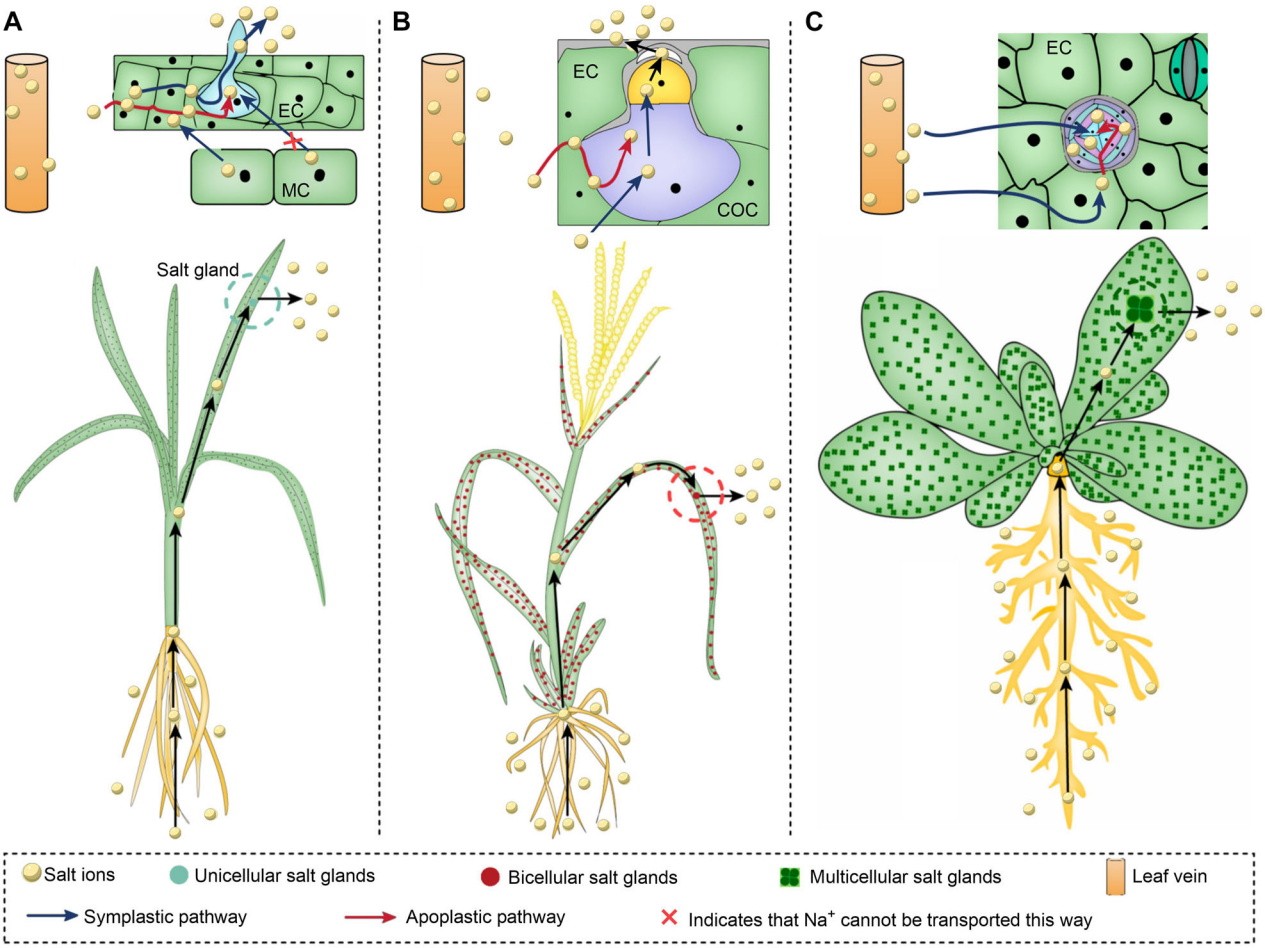
Schematic diagram of Na^+^ translocation from roots to leaf salt glands and its subsequent excretion in exo‐recretohalophytes In **(A)** *Oryza coarctata*, **(B)** *Sporobolus anglicus*, and **(C)** *Limonium bicolor*, Na^+^ is transported from the roots to the leaf salt glands and secreted from the plant, driven by transpiration. Abbreviations: COC, collecting cell; EC, epidermal cell; MC, mesophyll cell.

In high‐salinity environments, plants adjust the accumulation and distribution of Na^+^ ions among different organs by coordinating Na^+^ uptake, excretion, and long‐distance transport (Munns and Tester, 2008; Zhang et al., 2019a). Generally, Na^+^ is transported within plants via three primary pathways: the apoplastic pathway, the symplastic pathway, and the transmembrane pathway (Hasegawa, 2013). Driven by water flow, Na^+^ ions are transported from the root surface to vascular tissues via the apoplastic and symplastic pathways (Faiyue et al., 2010; Isayenkov and Maathuis, 2019). However, the influx of Na^+^ through the apoplastic pathway is constrained by hydrophobic barriers such as the Casparian strip in the endodermis and the exodermis. As a result, the primary route of Na^+^ entry into the vasculature remains unresolved (Gao et al., 2023). Once Na^+^ ions enter vascular tissues, they are transported upward through the xylem via the transpiration stream. In leaves, Na^+^ ions may be delivered to mesophyll or epidermal cells through either symplastic or apoplastic pathways (Figure 4).

In plants with unicellular salt glands, Na^+^ ions enter salt gland cells via both symplastic and apoplastic routes (Figure 4A) (Naidoo and Naidoo, 1999). Nevertheless, the absence of plasmodesmata between unicellular salt glands and mesophyll cells restricts Na^+^ transport. Under these conditions, Na^+^ ions can enter the salt gland symplastically from adjacent epidermal cells or apoplastically from mesophyll or epidermal cells (Figure 4A) (Koteyeva et al., 2022). Upon entering the salt gland, Na^+^ ions are directed to the subcellular membrane chamber of the partitioning membrane system and are subsequently secreted from the plant via membrane‐bound ion transporters (Figure 4A) (Koteyeva et al., 2022).

In plants with bicellular salt glands, Na^+^ ions also gain entry into salt gland cells via symplastic and apoplastic pathways (Figure 4B) (Naidoo and Naidoo, 1999). After entering the salt glands, Na^+^ ions can move between salt gland cells through apoplastic, symplastic, and transmembrane routes. In the apoplastic pathway, discontinuities in the cuticle enable limited diffusion of Na^+^ between cells through cuticular gaps. In the symplastic pathway, Na^+^ ions are transported through plasmodesmata that connect adjacent salt gland cells. In the transmembrane pathway, Na^+^ ions are shuttled across individual cells via asymmetric transport across the plasma membrane—entering the cell on one side and exiting on the other (Hou et al., 2022). Through these ways, Na^+^ ions are transported from the BC to the CC, before being expelled from the apex of the CC (Figure 4B) (Koteyeva et al., 2022).

In plants with multicellular salt glands, these cells are surrounded by a thick cuticle layer that restricts apoplastic transport. Therefore, Na^+^ ions can only enter multicellular salt glands from mesophyll or epidermal cells via the symplastic pathway (Ma and Yuan, 2023) (Figure 4C). After Na^+^ ions enter the salt gland, they move between salt gland cells via the apoplastic, symplastic, and transmembrane pathways. Na^+^ ions are thus transported from epidermal or mesophyll cells to the OCs, ICs, ACs, and SCs. Finally, Na^+^ ions are transferred from the SCs into the secretory chamber and secreted through the secretory pores (Figure 4C) (Yuan et al., 2015a). Additionally, the transport of Na^+^ ions from the roots to salt gland cells, as well as between the cells within the salt gland, is governed by ion transporters (Yuan et al., 2016a; Li et al., 2020). The genes involved in regulating Na^+^ transport from roots to salt gland cells and between salt gland cells are listed in Table S2.

### Transport of salt from salt glands to the extracellular space

The electrophysiological characteristics of plant salt gland biomembranes indicate that the ion transport system operates primarily via active transport. Early microelectrode measurements revealed that SCs maintain a metabolism‐dependent negative membrane potential (Hill, 1967; Hill and Hill, 1976), which is generated and maintained primarily by the plasma membrane H^+^‐ATPase. The resulting proton electrochemical gradient then drives the secondary active transport of Na^+^ and Cl^−^, ultimately enabling their excretion against a concentration gradient. Studies on the salt glands of *A. marina* further support the active nature of this process: Although ion concentrations in SCs are low, the declining ion concentration gradient from xylem‐associated cells to mesophyll cells and then to salt glands indicates the presence of active ion pumps in specific biomembranes, capable of loading and secreting ions against a concentration gradient (Shimony et al., 1973).

Three main hypotheses have been proposed to explain the mechanism of salt secretion by salt glands in exo‐recretohalophytes, each differing in the underlying cellular processes and driving forces. The osmotic mechanism hypothesis posits that plants actively transport Na^+^ ions into salt gland cells, leading to an increase in intracellular hydrostatic pressure. Once the pressure exceeds a certain threshold, the salt glands expel Na^+^ ions in the form of microscopic water droplets (Arisz et al., 1955; Lu et al., 2022). By contrast, the reverse pinocytosis hypothesis focuses on vesicular trafficking: It proposes that small cytoplasmic vesicles accumulate large amounts of Na^+^ ions, which are then excreted from the cell via the expulsion of small vesicles, opposite to the direction of typical pinocytosis (Shimony and Fahn, 1968; Wilson et al., 2017). The animal‐like fluid transport hypothesis postulates that Na^+^ ions move through the BCs of the salt gland, enter the SCs, and are actively transported to the extracellular space through ion transport carriers powered by ATP hydrolysis (Levering and Thomson, 1971; Zhao et al., 2023).

**Figure 5:**
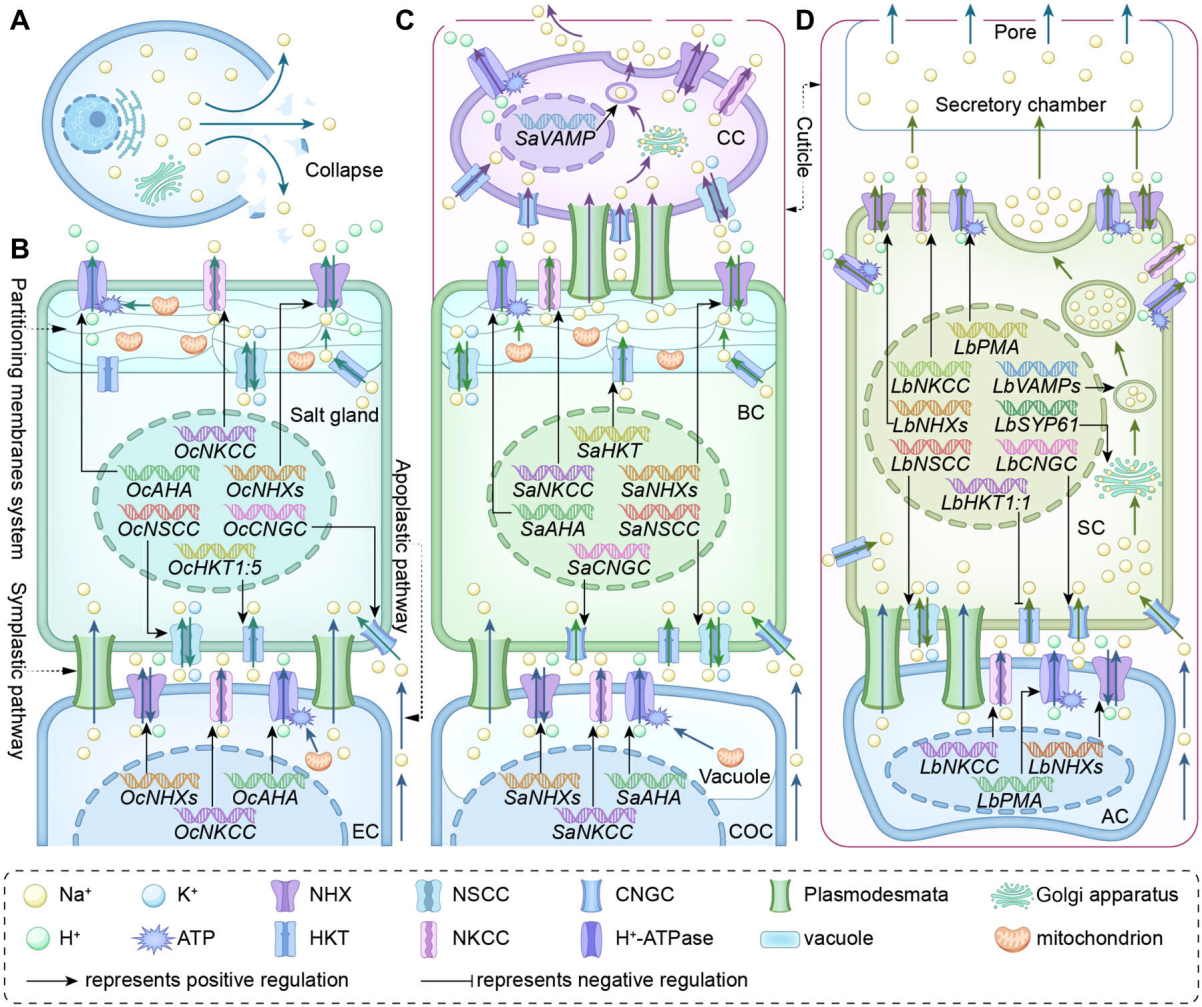
Salt secretion process and molecular regulatory mechanisms in plant salt glands This figure illustrates **(A**, **B)** the unicellular salt glands of *Oryza coarctata*, **(C)** the bicellular salt glands of *Sporobolus anglicus*, and **(D)** the multicellular salt glands of *Limonium bicolor*, depicting the salt secretion pathway whereby ions are taken up from adjacent cells, transported intracellularly within secretory cells, and ultimately secreted through the apical structure. Abbreviations: AC, accessory cell; BC, basal cell; CC, cap cell; COC, collecting cell; EC, epidermal cell; SC, secretory cell.

In plants with unicellular salt glands, both the osmotic mechanism hypothesis and the animal‐like fluid transport hypothesis have garnered empirical support, while the reverse pinocytosis hypothesis remains unsupported by experimental data. In species such as *O. coarctata*, upper and lower leaf epidermal salt glands show distinct secretion modes. Lower epidermal salt glands can collapse and rupture, releasing Na^+^ with the efflux of fluid—a process consistent with the osmotic mechanism (Figure 5A) (Flowers et al., 1990; Sengupta and Majumder, 2009). When external Na^+^ concentrations decrease, new salt glands can regenerate and resume Na^+^ secretion (Sengupta et al., 2008; Rajakani et al., 2019). By contrast, the upper epidermal salt glands can continuously excrete Na^+^ ions by a system similar to animal fluid transport (Sengupta et al., 2008). Within these cells, a partitioning membrane system connected to the apical plasma membrane is enriched with mitochondria, suggesting a role in transporting Na^+^ from the cytoplasm into this compartment (Figure 5B). The expanded membrane surface and high mitochondrial density are thought to support active ion transport by providing ample energy (Koteyeva et al., 2022). Mechanical scraping and RNA‐seq analysis of isolated unicellular salt glands identified transporter genes such as *Na* ^*+*^ */H* ^*+*^ *EXCHANGER 1* (*OcNHX1*) and *HIGH‐AFFINITY K* ^*+*^ *TRANSPORTER 1;5* (*OcHKT1;5*) as being expressed in salt gland cells (Rajakani et al., 2019). Subsequent studies determined that under salt treatment, expression of *SALT OVERLY SENSITIVE 1* (*OcSOS1*), *OcNHX1*, *OcHKT1;4*, and *H* ^*+*^ *‐ATPase* (*OcAHA*) is strongly upregulated in *O. coarctata* (Yong et al., 2022; Yun et al., 2025), providing molecular evidence that Na^+^ ions are secreted via ion transporters and channels (Figure 5B).

In plants with bicellular salt glands, the osmotic mechanism hypothesis, the reverse pinocytosis hypothesis, and the animal‐like fluid transport hypothesis are all supported by molecular evidence. In bicellular salt glands, such as those found in *D. spicata*, the double‐membrane (plasma membrane and vacuolar membrane) structure at the top of the COCs disappears, exposing the vacuolar membrane directly to the extracellular matrix and establishing it as the sole barrier for ion efflux (Figure 5C). This vacuolar membrane forms a valve‐like structure that may regulate Na^+^ entry into the extracellular matrix. Additionally, BCs show two distinct shapes, narrow and wide, which are thought to be associated with mechanical compression or expansion of the plasma membrane, respectively, due to changes in osmotic pressure. This switch between compression and expansion may alter the morphology of extracellular channels, thereby modulating Na^+^ ion transport from the vacuole of COCs to BCs (Semenova et al., 2010). These structural features provide support for the osmotic mechanism hypothesis. In *Spartina alterniflora* Loisel., laser microdissection was used to isolate bicellular salt glands for RNA‐seq analysis. This approach identified *SaVAMP*, encoding a vesicle‐associated membrane protein (VAMP), as being involved in salt secretion, providing direct molecular evidence supporting the reverse pinocytosis hypothesis in bicellular salt glands (Figure 5C) (Liu, 2011). In addition, inhibition of Na^+^ secretion by the Na^+^‐ATPase inhibitor ouabain in *Chloris gayana* Kunth supports a role for active ion transport (Kobayashi et al., 2007). In *Cynodon dactylon* (L.) Persoon, the expression of *CdSOS1* and *CdNHX* is upregulated under salt stress (Roy and Chakraborty, 2018). Moreover, structural studies in *S. anglicus* and *Urochondra setulosa* (Trin.) C.E. Hubb. revealed that the BCs of their bicellular salt glands possess a partitioning membrane system similar to that of unicellular salt glands. This system is enriched with mitochondria and ion transporters, suggesting a role in Na^+^ transport from basal to CCs (Koteyeva et al., 2022), supporting the functional relevance of ion transporters in salt gland secretion (Figure 5C).

In plants with multicellular salt glands, the reverse pinocytosis hypothesis and the animal‐like fluid transport hypothesis are supported by structural, physiological, and molecular evidence; the osmotic mechanism hypothesis appears unlikely, as no cell collapse, fragmentation, or valve‐like structures have been observed in multicellular salt glands (Figure 5D). This absence suggests that a simple osmotic mechanism cannot fully account for salt secretion in multicellular salt glands (Yuan et al., 2015a). In *T. chinensis*, treatment with a radioactive isotope of rubidium (Rb^+^) resulted in electron‐induced damage to vesicles observed under electron microscopy (Thomson et al., 1969). Similarly, transmission electron microscopy of ultrathin sections of *L. bicolor* leaves revealed the fusion of small vesicles with the plasma membrane (Yuan et al., 2015b). Further RNA‐seq analysis of *L. bicolor* plants irrigated with 200 mM NaCl identified 18 significantly upregulated genes encoding vesicle transport‐related proteins, implying a key role for vesicle trafficking in salt secretion (Yuan et al., 2016b). Treatment with 200 μg/mL brefeldin A (BFA), a vesicle transport‐specific inhibitor, disrupted Golgi apparatus function, significantly inhibited acid phosphatase activity, and diminished salt secretion in *L. bicolor* (Lu et al., 2020). In addition, treatment with melatonin, which promotes salt secretion in *L. bicolor*, significantly upregulated the expression of the vesicle transport‐related genes *LbVAMP721*, *LbVAMP27*, and *LbVAMP121*, further implicating vesicle trafficking in this mechanism (Figure 5D) (Li et al., 2020). To functionally validate the role of vesicle transport, *SYNTAXIN 61* (*LbSYP61*), encoding a trans‐Golgi‐localized SNARE protein, was silenced, resulting in reduced salt secretion and confirming the importance of vesicle transport (Figure 5D) (Lu et al., 2020). In addition, proteomic analysis of salt gland secretions in *L. bicolor* via SDS‐PAGE and mass spectrometry identified vesicle transport‐associated proteins, including Golgi marker enzymes (e.g., glycosyltransferases) and cargo proteins (Lu et al., 2022). These findings collectively support the conclusion that multicellular salt glands likely secrete salt via a vesicle‐mediated mechanism consistent with reverse pinocytosis.

Several lines of physiological evidence support the animal‐like fluid transport model for multicellular salt glands. In *A. germinans*, treatment with the H^+^‐ATPase inhibitors vanadate or sodium diethyldithiocarbamate (DIDS) significantly inhibited salt secretion (Dschida et al., 1992; Balsamo et al., 1995). In *T. chinensis* and *L. bicolor*, application of the vertebrate Na^+^/K^+^‐ATPase inhibitor ouabain or the Na^+^: K^+^: Cl^−^ cotransporter (NKCC) inhibitor bumetanide also markedly diminished salt secretion (Ma et al., 2011; Yang et al., 2012). In *Avicennia officinalis* L., the acidic nature of the membrane periphery of secretory organelles and the peripheral cuticle indicates that H^+^‐ATPase activity generates a transmembrane electrochemical gradient to drive the activities of other ion transporters (Tan et al., 2010). At the molecular level, treatment of *A. marina* with nitric oxide (NO), hydrogen sulfide (H_2_S), or 400 mM NaCl upregulated the expression of *AmAHA1*, *AmSOS1*, and *AmNHX1*, thereby enhancing salt gland secretion (Chen et al., 2010; Wei et al., 2022; Guo et al., 2023). Additionally, expression of *AmbHLH091* was significantly induced under salt stress, where it promotes Na^+^ transport by positively regulating AmSOS1 expression. AmNAC035 interacts with AmbHLH091 and represses its transcriptional activity, thus fine‑tuning *AmSOS1* expression (Tang et al., 2025). In *L. bicolor*, exogenous melatonin treatment significantly increased the salt secretion rate of salt glands and the expression levels of genes encoding ion transporters (*LbSOS1* and *LbNHX1*), indicating the involvement of ion channels in salt efflux (Ding et al., 2010). The H^+^‐ATPase PLASMA MEMBRANE PROTON ATPASE (LbPMA) was highly abundant in salt glands, based on immunohistochemical localization, with its levels increasing under higher NaCl concentrations. Functional studies revealed that LbPMA and LbSOS1 act synergistically; LbPMA establishes a proton gradient that powers LbSOS1‐mediated Na^+^ extrusion against the concentration gradient (Figure 5D) (Ding et al., 2010). Furthermore, expression of *NON‐SELECTIVE CATIONIC CHANNEL* (*LbNSCC*) and *CYCLIC NUCLEOTIDE‐GATED ION CHANNEL* (*LbCNGC*) correlated positively with salt secretion capacity (Yuan et al., 2016a; 2016b). A high‐affinity potassium transporter gene, *LbHKT1*;*1*, was also identified in *L. bicolor*. Despite lacking K^+^ transport activity, LbHKT1;1 negatively regulates salt secretion, as demonstrated by Na^+^ secretion rates following the VIGS‐mediated silencing of this gene (Zhu et al., 2025b). Together, these physiological, structural, and molecular findings support the hypothesis that multicellular salt glands can secrete salt via mechanisms analogous to the animal fluid transport system (Figure 5D).

The secretory activity of salt glands is influenced by external environmental factors and internal genetic regulation. The regulatory genes constitute a complex network that synergistically governs salt secretion (Figure 5). Genes associated with salt secretion from salt glands to the extracellular space are listed in Table S3.

The osmotic, reverse pinocytosis, and animal‐like fluid transport hypotheses have been proposed to explain how exo‐recretohalophytes secrete excess salts. A key premise common to these models is the requirement for metabolic energy, often in the form of ATP, to initiate the secretory process (Naidoo and Naidoo, 1999; Ma and Yuan, 2023). However, emerging evidence indicates that salt gland secretion likely involves a complex, synergistic mechanism that integrates elements from multiple hypotheses, rather than being fully explained by any one alone.

As shown in Table S4, the osmotic hypothesis—primarily applicable to unicellular and bicellular salt glands—identifies hydrostatic pressure as the core driving force. Its validity is supported by the collapse and rupture of unicellular salt glands under osmotic stress, as well as the osmosis‐driven morphological transitions in bicellular salt glands (Sengupta and Majumder, 2009; Semenova et al., 2010). However, the osmotic mechanism generally lacks selectivity. In contrast, the reverse pinocytosis hypothesis and the animal‐like fluid transport hypothesis propose mechanisms that are more selective (Gao et al., 2017; Rajakani et al., 2019). The reverse pinocytosis hypothesis implicates vesicular transport in secretion, which is supported by evidence such as the involvement of the vesicle‐associated protein SaVAMP in bicellular glands (Liu, 2011), reduced secretion in *L. bicolor* upon treatment with the vesicular transport inhibitor BFA (Lu et al., 2020), and the identification of vesicle‐related proteins among the secretory proteome of multicellular glands (Lu et al., 2022). The animal‐like fluid transport hypothesis suggests a complex regulatory hierarchy, applicable to all salt gland types. It posits that active transport via a series of ion transporters and pumps (e.g., OcNHX1, OcHKT1;5, LbPMA, LbNSCC, and LbCNGC) establishes ion gradients that drive water osmosis. This mechanism is strongly supported by the inhibitory effects of various ATPase and transporter inhibitors (Ding et al., 2010; Yuan et al., 2016a; 2016b; Rajakani et al., 2019), while the enrichment of mitochondria near the partition membranes of unicellular and bicellular salt glands (Koteyeva et al., 2022) further provides structural support for this energy‐dependent process.

Collectively, these findings argue against a single, universal mechanism. Salt gland secretion more likely involves a dynamic interplay of osmotic, vesicular, and animal‐like fluid transport processes, the balance of which is modulated by the plant's physiological state and development.

### Salt secretion of salt glands is affected by environmental conditions and phytohormones

Different exo‐recretohalophyte species show distinct salt secretion rates over a 24 h period (Atkinson et al., 1967). For example, the Na^+^ secretion rate of a single salt gland in *L. bicolor* leaves can reach approximately 600 pmol cm^−2^ s^−1^ (Yuan et al., 2022), whereas that of *A. marina* can reach at most 164 pmol cm^−2^ s^−1^ (Wei et al., 2022). These rates also show diurnal fluctuations. Studies in species such as *Reaumuria trigyna* Maxim. and *A. marina* indicate that salt secretion is influenced by the plant's circadian clock (Scholander et al., 1962; Xue and Wang, 2008). In *R. trigyna*, the salt secretion rate is higher in the morning than in the afternoon, and higher during the day than at night. Conversely, in *A. marina*, the salt secretion rate is greater at night than during the day (Wang and Liu, 2019). Moreover, salt secretion capacity can differ among various tissues and developmental stages within the same species. For example, the basal epidermis of the petiole in *L. bicolor* contains a special bead‐like salt gland composed of more than sixteen cells, which shows a higher secretion capacity than the typical salt glands present on the leaf surface (Zhao et al., 2023).

Ions regulate salt gland secretion in three main respects: Concentration dependence, ion‐specific differences, and selective modulation by specific ion types. First, in *L. sinense*, the secretion rate of salt glands is positively correlated with the external salt concentration; indeed, the Na^+^ concentration in secretions increases with a higher Na^+^ ion concentration in the culture medium (Zhao et al., 1999; Ding et al., 2009). Second, also in *L. sinense*, the secretion rates of different ions vary; for example, Na^+^ ions are secreted at a higher rate than K^+^ (Ding et al., 2009). Third, in *Limonium vulgare* Mill., salt glands show relatively low ion selectivity. When non‐natural and non‐essential ions such as lithium (Li^+^), Rb^+^, and cesium (Cs^+^) are added to the culture medium, these ions can also be detected in salt gland secretions (Yuan et al., 2015a). In addition, studies on *L. bicolor* have shown that treatment with 20 mM Ca^2+^ was shown to enhance salt secretion (Ding et al., 2010), whereas treatment with bromide (Br^−^), sulfate (SO_4_^2−^), and bicarbonate (HCO_3_^−^) inhibited Na^+^ secretion, revealing that specific ions exert regulatory effects on the secretion rate (Yang et al., 2012).

Phytohormones are pivotal signaling molecules that regulate salt secretion in *L. bicolor*. MeJA exerts concentration‐dependent effects: Low concentrations of MeJA (< 0.03 mM) do not significantly affect the Na^+^ secretion rate per gland, whereas higher concentrations (> 0.03 mM) significantly suppress it (Yuan et al., 2018). In addition, treatment with 5 μM melatonin enhances the secretion rate of individual salt glands under 300 mM NaCl stress (Li et al., 2020). Similarly, ABA promotes salt secretion; under 300 mM NaCl stress, 10 μM ABA significantly increased single‐gland secretion volume and reduced the Na^+^ content in *L. bicolor* seedlings (Zhu et al., 2024b).

Abiotic environmental factors, including temperature, humidity, and light intensity, modulate the secretion capacity of salt glands (Gorham, 1987; Hou et al., 2022). As the temperature increases, the salt excretion rate increases; for example, the rate at 39°C is approximately three times that at 32°C and about 20 times that at 19°C. Higher humidity and light intensity also promote salt gland secretion (Ppllak and Waisel, 1979; Hou et al., 2022).

### Diversity of salt gland secretion

In addition to their primary role in secreting Na^+^ and Cl^−^, salt glands in various exo‐recretohalophytes can also secrete a range of other inorganic ions and organic solutes, highlighting their functional versatility.

Salt glands show a broad capacity for inorganic ion secretion, extruding various cations and anions across different species. For instance, the unicellular salt glands of *O. coarctata* and the bicellular salt glands of *S. anglicus* and *U. setulosa* secrete K^+^ (Koteyeva et al., 2022); the bicellular salt glands of *C. gayana* secrete Li^+^ (Yaokawa et al., 2025); and those of *Sporobolus virginicus* (L.) Kunth secrete Ca^2+^ and Mg^2+^ (Naidoo and Naidoo, 1998). Notably, some species show a particularly wide secretory spectrum. For instance, the multicellular salt glands of *R. trigyna* secrete Na^+^, K^+^, Ca^2+^, Mg^2+^, SO₄^2−^, and Cl^−^ (Xue and Wang, 2008).

Salt gland function is not limited to inorganic ion excretion. For instance, salt glands in *Tamarix* species secrete small amounts of organic solutes such as proline (Zhang and Zhang, 2015), while studies of *L. bicolor* have identified numerous secreted proteins and peptides (Lu et al., 2022). These observations suggest that salt glands contribute not only to ion homeostasis but also to the active transport and extracellular release of organic molecules.

## DISCUSSION AND PERSPECTIVES

Exo‐recretohalophytes possessing salt glands have considerable potential for application. However, significant knowledge gaps persist in salt gland research. This section discusses the possible origins and evolutionary relationships with other epidermal structures, as well as their application in crops improvement. Building on this discussion, we then outline five key unresolved questions in the field.

### The origin of salt glands and their evolutionary relationship with hydathodes, trichomes, and oil glands

The evolutionary origins of salt glands are polyphyletic and complex. Structural differences in salt glands across various plant groups suggest that they originated independently. Studies indicate that in angiosperms alone, salt glands have evolved independently at least 12 times (Dassanayake and Larkin, 2017). The independent emergence of morphologically similar salt‐secreting structures across distantly related lineages is a textbook case of convergent evolution, reflecting similar adaptive strategies of different plant groups to high‐salinity environments.

Salt glands, hydathodes, trichomes, and oil glands are all considered homologous structures. According to the prevailing hypothesis, salt glands likely originated from an ancestral structure similar to the hydathodes of ferns, a view supported by similarities in both structure and function (Lüttge, 2019). First, both salt glands and fern hydathodes are specialized epidermal structures, showing a continuum of morphological features during evolution (Lüttge, 2019). Second, the distribution pattern of hydathodes on the leaf surface in ferns resembles the regularly spaced arrangement of salt glands in the typical exo‐recretohalophytes *L. bicolor* (Cerutti et al., 2019; Mishra et al., 2021; Mehltreter et al., 2022). Although both structures consist of SC clusters tightly enclosed by surrounding cells, salt glands display greater cellular complexity, typically comprising four distinct cell types (Mehltreter et al., 2022; Yang et al., 2024). Functionally, hydathodes primarily excrete excess water driven by root pressure (Paauw et al., 2023), whereas in some fern species (e.g., *Campyloneurum brevifolium* (Lodd. ex Link) Link, *Pityrogramma calomelanos* (L.) Link, and *Pyrrosia polydactylos* (Hance) Ching), hydathodes play dual roles—both draining water and secreting specific ions, including Na^+^ (Mehltreter et al., 2022). These structural and functional differences indicate that salt glands are a more highly specialized evolutionary product compared to hydathodes.

There is also a significant connection between salt glands and trichomes. Both salt glands and trichomes originate from MESCs and have three morphological types: Unicellular, bicellular, and multicellular (Han et al., 2022a). They are also more densely distributed on the abaxial leaf epidermis (Xing et al., 2012; Zhao et al., 2022). The spatial arrangement of salt glands in *L. bicolor* and trichomes in *Arabidopsis* both show a regular, spaced pattern (Mishra et al., 2021). Functionally, trichomes are categorized into glandular trichomes, which possess secretory capability, and non‐glandular trichomes, which lack it. Glandular trichomes participate in defense against herbivores and abiotic stress by secreting secondary metabolites (Karabourniotis et al., 2020; Schuurink and Tissier, 2020; Wang et al., 2020). This secretory mechanism shares similarities to the salt‐secreting function of salt glands.

Furthermore, salt glands and oil glands share similarities in both origin and function: Both originate from MESCs (Bai et al., 2020), are embedded in the epidermal tissue, and show a regular, spaced pattern (Mishra et al., 2021; Wang et al., 2024a). Oil glands secrete essential oil components—such as monoterpenes, fatty acids, and flavonoids—to combat pathogens and pests (Xie et al., 2024; Wang et al., 2024a).

In summary, salt glands, hydathodes, trichomes, and oil glands show broad similarities in spatial distribution, morphological structure, developmental origin, and partial functions. These shared characteristics support their common origin from multipotent epidermal ancestral structures. Their morphological and functional diversification represents an evolutionary outcome of plants’ adaptation to different ecological environments and selective pressures over the long term.

### Applications for exo‐recretohalophytes with salt glands in crops

Exo‐recretohalophytes with salt glands represent valuable genetic resources for improving salinity tolerance. Their core value lies not only in their potential domestication for direct exploitation of salt tolerance but also in integrating their salt gland structures and related genes into crops, offering novel strategies to address the escalating challenge of soil salinization.

Direct domestication is the most straightforward strategy. Differential domestication objectives can be tailored to the specific biology of different species. For instance, this includes using *O. coarctata* as a genetic donor to introduce unicellular salt gland traits into cultivated rice; enhancing the salt tolerance and biomass of species with bicellular salt glands like *B. gracilis* and *D. spicata* to develop pasture grasses specifically for saline–alkaline lands (Al‐Shorepy et al., 2010; Ji et al., 2022); and for species with multicellular salt glands, pursuing multiple domestication trajectories. This could involve increasing target product content in species with medicinal value, such as *T. chinensis* (Ding and Zhang, 2024), *A. marina* (Chen et al., 2022), *L. bicolor* (Wang and Duan, 2008; Weng, 2008; Zhu and Yuan, 2012), and *L. aureum* (Tian et al., 2010); optimizing ornamental traits in species like *L. aureum* and *L. sinuatum* (Li et al., 2010) or improving biomass and protein content in species like *L. gmelinii* (He, 2014) for forage cultivar development. Notably, many species with multicellular salt glands (e.g., some species in the genus *Limonium*) also possess perennial and natural overwintering characteristics, which significantly enhance their domestication value. This perennial growth habit reduces the need for annual replanting, enhancing their suitability for marginal lands. Furthermore, their subterranean storage organs ensure continuity in biomass or active ingredient accumulation, providing a core biological foundation for sustainable, large‐scale production.

However, traditional domestication processes are often lengthy. To accelerate the introduction of the superior salt gland trait into existing cultivated crops, distant hybridization and genetic engineering offer more efficient alternatives. For example, *O. coarctata* with unicellular salt glands can be used as a donor parent for hybridization with cultivated rice to introduce the salt gland structure into the cultivated rice system (Shanmugham et al., 2025). Similarly, monocotyledonous exo‐recretohalophytes with bicellular salt glands can serve as maternal parents for distant hybridization with cereal crops to achieve the introduction of bicellular salt glands. For multicellular salt glands, primarily found in dicots, interspecific hybridization techniques can be used to introduce them into related crops. Genetic engineering provides a complementary strategy: By identifying and introducing key genes regulating salt gland development, it is promising to directly construct functional salt glands in target crops.

Additionally, introducing key genes regulating salt gland development and salt secretion into crops via genetic engineering represents a promising biotechnological approach to enhancing salt tolerance. This approach is particularly valuable for genes lacking homologs in the genomes of existing crops. Furthermore, based on the systemic conservation among plant taxa, utilizing salt gland‐regulatory genes from monocots to improve monocot crops, or applying related genes from dicots to dicot crops, is more conducive to maintaining the functional compatibility of the genes. For instance, the heterologous expression of genes from *L. bicolor* in *Arabidopsis* such as *LbTTG1* (Yuan et al., 2019), *LbSAD2* (Xu et al., 2020), *LbHLH* (Wang et al., 2021), *LbMYB48* (Han et al., 2022b), *LbRZF1* (Yang et al., 2024), *LbRSG* (Zhou et al., 2024), *LbMYB368* (Zhu et al., 2025a), and *Lb1G04202* (Wang et al., 2022b) significantly enhanced salt tolerance at both seedling and mature plant stages. These findings fully demonstrate the potential value of salt gland‐regulatory genes in future crop improvement.

Nevertheless, both hybridization and genetic engineering, especially the latter, face severe challenges when attempting to construct a fully functional salt gland in crops. Distant hybridization must continuously address issues such as reproductive barriers and complex segregation of traits in progeny. The successful construction of salt glands via genetic engineering is constrained by at least two major limitations: First, the complete set of key genes regulating salt gland initiation, differentiation, and maturation, along with their interaction networks, has not been fully elucidated. Second, there is a current lack of promoter systems capable of driving the expression of these genes in specific epidermal cells in a strict spatiotemporal pattern. This necessitates future research to develop composite or synthetic promoters integrating tissue specificity and developmental stage specificity to achieve precise programming of salt gland development.

Beyond the technical operational level, introducing an additional and energy‐consuming salt‐secreting organ into crop plants requires prudent evaluation from the perspectives of energetic cost and overall plant physiological compatibility. The operation of salt glands depends on ATP energy supply. Theoretical analysis suggests that under moderate‐to‐severe and persistent salt stress conditions, the energy cost of driving ion efflux might be lower than the cost of synthesizing and accumulating compatible solutes to maintain cellular homeostasis. In such scenarios, conferring salt secretion capacity to crops may offer an energetic advantage (Chen et al., 2010; Ding et al., 2010; Munns et al., 2020). Conversely, under mild or intermittent salt stress, this approach might lead to unnecessary growth inhibition. More complex is the issue of systemic compatibility: Salt stress itself disrupts ion homeostasis in sensitive plant cells, for example, by causing membrane potential depolarization (Wegner et al., 2011; Hamam et al., 2019), interfering with K^+^/Na^+^ balance (Tester and Davenport, 2003; Page and Di Cera, 2006), and inhibiting plasma membrane H^+^‐ATPase activity (Ibrahimova et al., 2021; Hualpa‐Ramírez et al., 2024). It remains unclear whether such disruptions would interfere with the ion transport function of newly introduced salt glands. A reasonable hypothesis is that if the development and functional activation of salt glands can be rapidly established before or simultaneously with salt stress, salt secretion through salt glands may alleviate intracellular ion imbalance and enhance crop salt tolerance. This hypothesis requires experimental validation.

In summary, despite the multifaceted challenges associated with harnessing the genetic resources of exo‐recretohalophytes with salt glands through domestication, distant hybridization, and genetic engineering, salt glands retain significant potential for enhancing crop salt tolerance. Their application holds particular theoretical and practical value for improving crop adaptability in moderate‐to‐severe saline–alkaline environments.

### Future prospects for salt gland research

The initiation and regulation of salt gland development represent a complex biological process. Recent studies have uncovered key genes involved in this regulatory network, advancing our understanding of how salt glands are formed and facilitate the excretion of excess salt ions from plant tissues. Despite these advances, significant knowledge gaps remain, hindering efforts to enhance salt gland function for improved salt stress tolerance or to engineer *de novo* salt glands in non‐halophytic crops. The main unresolved questions include the following:

I. Identifying the fate‐determining genes for salt gland differentiation. A core objective in the study of salt gland development is to identify fate‐determining genes that guide the differentiation of MESCs into salt gland cells. Although existing studies have discovered that some genes highly similar to trichome development genes are expressed in the early stages of salt gland development, the key genes that directly regulate salt gland differentiation have not yet been clearly identified.

II. Understanding the mechanisms governing salt gland morphogenesis. Within the same species (e.g., *L. bicolor*), salt glands with distinct morphological features and varying cell numbers can arise in different tissues, such as the leaf surface and the petiole base (Zhao et al., 2023). However, our current understanding of the developmental programs underlying these specialized salt glands remains limited. In particular, it is not yet clear whether salt gland morphogenesis in different tissues is governed by distinct, cell type‐specific regulatory signaling pathways.

III. Clarifying the homology between salt glands and other specialized epidermal structures. Previous studies have indicated that salt glands may be homologous to other MESC‐derived specialized epidermal structures, such as hydathodes, trichomes, and oil glands (Lüttge, 2019; Mehltreter et al., 2022; Han et al., 2022a; Wang et al., 2024a). However, the evolutionary continuity among these specialized epidermal structures cannot be verified due to the lack of known intermediate transitional forms that connect them. More critically, the molecular mechanisms governing the differentiation of MESCs into these specialized epidermal structures remain unclear, further preventing us from confirming the homology between salt glands and other specialized epidermal structures.

IV. Elucidating the salt secretion mechanism of salt glands. Current studies speculate that salt glands may mainly rely on the synergy of reverse pinocytosis and the animal‐like fluid transport system for salt secretion. However, there are clear differences among different types of halophytes, and different salt secretion mechanisms may coexist in the same halophyte species. In particular, key genes involved in salt secretion have yet to be identified in any type of exo‐recretohalophyte, and the relative contribution of these different mechanisms to salt secretion and their spatiotemporal coordination are also unclear.

V. Overcoming technical challenges to construct salt glands in crop species. The formation of functional salt glands in crop species is a core goal for the future application of exo‐recretohalophytes in agriculture. However, salt gland development requires precise coordination of multiple genes (stage‐specific temporal expression and spatial restriction to specific epidermal cells). Even if key genes underlying salt gland development are identified, a key technical bottleneck remains: There is currently a lack of efficient promoter systems capable of conferring both tissue specificity and developmental stage precision, thereby hindering strict spatiotemporal control of transcriptional regulation for target genes.

## CONFLICTS OF INTEREST

The authors declare no conflicts of interest.

## AUTHOR CONTRIBUTIONS

G. H. proposed the outline of the review. L. W., J. X., Y. Z., C. Y., and H. F. wrote the review. C. S., W. Z., J. Q., X. Z., B. W., J. Z., and G. H. revised the review. All authors read and approved the final manuscript.

## Supporting information

Additional Supporting Information may be found online in the supporting information tab for this article: http://onlinelibrary.wiley.com/doi/10.1111/jipb.70141/suppinfo

**Table S1.** Genes associated with salt gland development
**Table S2.** Genes associated with the salt secretion pathway from mesophyll or epidermal cells to salt glands
**Table S3.** Genes associated with salt secretion from salt glands to extracellular space
**Table S4.** Comparison of the three major hypotheses for salt secretion by salt glands
