# Supplementary material for: Salt glands in exo‐recretohalophytes: Development, physiological functions, and prospects for improving crop salt tolerance
Source: J Integr Plant Biol. 2026 Jan 19;68(8):2368–87. doi: 10.1111/jipb.70141 (PMC13446620; doi:10.1111/jipb.70141)
Supplement: Supplementary file 1 [file JIPB-68-2368-s001.docx]

**Supplementary Table 1 Genes associated with salt gland development**

| Genes | Type | Regulation | Species | References |
| --- | --- | --- | --- | --- |
| *LbHLH* | bHLH | - | *L. bicolor* | (Yuan et al., 2022) |
| *Lb1G07934* | bHLH | - | *L. bicolor* | (Wang et al., 2024c) |
| *LbMAPK2* | MAPK | + | *L. bicolor* | (Zhang et al., 2023a) |
| *LbMAPK10* | MAPK | + | *L. bicolor* | (Zhang et al., 2023a) |
| *LbWRKY10* | WRKY | + | *L. bicolor* | (Zhu et al., 2024b) |
| *LbAP2/ERF* | AP2/ERF32 | + | *L. bicolor* | (Jiang et al., 2024) |
| *LbGIS2* | C2H2-ZFPs | + | *L. bicolor* | (Zhao et al., 2024) |
| *LbRZF1* | RING-HC | + | *L. bicolor* | (Yang et al., 2024) |
| *LbCAT2* | CAT | + | *L. bicolor* | (Yang et al., 2024) |
| *LbMYB48* | R1-MYB | + | *L. bicolor* | (Han et al., 2022b) |
| *LbMYB113* | R2R3-MYB | + | *L. bicolor* | (Yang et al., 2024) |
| *LBMYB368* | R2R3-MYB | + | *L. bicolor* | (Zhu et al., 2025) |
| *LbCPC* | R3-MYB | - | *L. bicolor* | (Zou et al., 2023) |
| *LbTRY* | R3-MYB | - | *L. bicolor* | (Zhao et al., 2024) |
| *BtRanscript_153392* | lncRNAs | + | *L. bicolor* | (Wang et al., 2024c) |
| *LbRSG* | Transmembrane protein | + | *L. bicolor* | (Zhou et al., 2024) |
| *LbNAC4* | NAC | + | *L. bicolor* | (Zhao et al., 2023) |
| *LbNAC54* | NAC | + | *L. bicolor* | (Zhang et al., 2024a) |
| *LbTTG1* | WD40 repeat-like superfamily | - | *L. bicolor* | (Yuan et al., 2022) |
| *LbbZIP28* | bZIP | - | *L. bicolor* | (Meng et al., 2024) |
| *LbSAD2* | Importin-β protein | + | *L. bicolor* | (Ma et al., 2025) |
| *Lb1G04794* | atypical | + | *L. bicolor* | (Jiao et al., 2022) |
| *Lb3G16832* | atypical | + | *L. bicolor* | (Zhou et al., 2024) |
| *Lb7G34824* | atypical | - | *L. bicolor* | (Zhao et al., 2024) |
| *Lb2G12567* | atypical | + | *L. bicolor* | (Ma et al., 2025) |
| *Lb2G12077* | atypical | - | *L. bicolor* | (Ma et al., 2025) |

**Supplementary Table 2 Genes associated with salt secretion pathway from mesophyll or epidermal cells to salt glands**

| Genes | Encoded proteins | Function | Species | Reference |
| --- | --- | --- | --- | --- |
| *AmAHA1* | H^+^-ATPase | Reverse concentration gradient transmembrane transfer of energy released from hydrolyzed ATP to H^+^ | *A. marina* | (Chen et al., 2010) |
| *LbPMA* | H^+^-ATPase | Reverse concentration gradient transmembrane transfer of energy released from hydrolyzed ATP to H^+^ | *L. bicolor* | (Li et al., 2020) |
| *OcAHA* | H^+^-ATPase | Reverse concentration gradient transmembrane transfer of energy released from hydrolyzed ATP to H^+^ | *O. coarctata* | (Yong et al., 2022) |
| *CdNHX* | Na^+^/H^+^ reverse transporter | Pump Na^+^out of the cell or store it in vacuoles | *C. dactylon* | (Roy and Chakraborty, 2018) |
| *OcNHX1* | Na^+^/H^+^ reverse transporter | Pump Na^+^out of the cell or store it in vacuoles | *O. coarctata* | (Rajakani et al., 2019) |
| *LbNHX1* | Na^+^/H^+^ reverse transporter | Pump Na^+^out of the cell or store it in vacuoles | *L. bicolor* | (Li et al., 2020) |
| *AmNHX1* | Na^+^/H^+^ reverse transporter | Pump Na^+^out of the cell or store it in vacuoles | *A. marina* | (Wei et al., 2022) |
| *OcSOS1* | Na^+^/H^+^ reverse transporter | Pump Na^+^out of the cell or store it in vacuoles | *O. coarctata* | (Roy and Chakraborty, 2018) |
| *LbSOS1* | Na^+^/H^+^ reverse transporter | Pump Na^+^out of the cell or store it in vacuoles | *L. bicolor* | (Li et al., 2020) |
| *CdSOS1* | Na^+^/H^+^ reverse transporter | Pump Na^+^out of the cell or store it in vacuoles | *C. dactylon* | (Yong et al., 2022) |
| *AmSOS1* | Na^+^/H^+^ reverse transporter | Pump Na^+^out of the cell or store it in vacuoles | *A. marina* | (Tang et al., 2025) |
| *OcHKT1;5* | High affinity potassium transporter | Pump Na^+^ into cells | *O. coarctata* | (Rajakani et al., 2019) |
| *LbHKT1*;*1* | High affinity potassium transporter | Pump Na^+^ into cells | *L. bicolor* | (Zhu et al., 2025b) |
| *LbNKCC* | Na^+^: K^+^: Cl^-^ cotransporter | Pump Na^+^out of the cell | *L. bicolor* | (Yang et al., 2012) |
| *LbNSCC* | non-selective cationic channel | Na^+^ is transported into the cell while K^+^ is transported out of the cell | *L. bicolor* | (Yuan et al., 2016a) |
| *LbCNGC* | cyclic nucleotide-gated cation channel | Pump Na^+^ into cells | *L. bicolor* | (Yuan et al., 2016a) |

**Supplementary Table 3 Genes associated with salt secretion from salt glands to extracellular**

| Genes | Ecoded proteins | Function | Species | Reference |
| --- | --- | --- | --- | --- |
| *SaVAMP* | Vesicle transport related proteins | Participate in vesicle transport | *S. alterniflora* | (Liu, 2011) |
| *LbVAP27* | Vesicle transport related proteins | Participate in vesicle transport | *L. bicolor* | (Li et al., 2020) |
| *LbVAMP121* | Vesicle transport related proteins | Participate in vesicle transport | *L. bicolor* | (Li et al., 2020) |
| *LbVAMP721* | Vesicle transport related proteins | Participate in vesicle transport | *L. bicolor* | (Li et al., 2020) |
| *LbSYP61* | SNARE | Participate in exocytosis to expel Na^+^from cells | *L. bicolor* | (Lu et al., 2020) |
| *AmAHA1* | H^+^-ATPase | Reverse concentration gradient transmembrane transfer of energy released from hydrolyzed ATP to H^+^ | *A. marina* | (Chen et al., 2010) |
| *LbPMA* | H^+^-ATPase | Reverse concentration gradient transmembrane transfer of energy released from hydrolyzed ATP to H^+^ | *L. bicolor* | (Li et al., 2020) |
| *OcAHA* | H^+^-ATPase | Reverse concentration gradient transmembrane transfer of energy released from hydrolyzed ATP to H^+^ | *O. coarctata* | (Yong et al., 2022) |
| *CdNHX* | Na^+^/H^+^ reverse transporter | Pump Na^+^out of the cell or store it in vacuoles | *C. dactylon* | (Roy and Chakraborty, 2018) |
| *OcNHX1* | Na^+^/H^+^ reverse transporter | Pump Na^+^out of the cell or store it in vacuoles | *O. coarctata* | (Rajakani et al., 2019) |
| *LbNHX1* | Na^+^/H^+^ reverse transporter | Pump Na^+^out of the cell or store it in vacuoles | *L. bicolor* | (Li et al., 2020) |
| *AmNHX1* | Na^+^/H^+^ reverse transporter | Pump Na^+^out of the cell or store it in vacuoles | *A. marina* | (Wei et al., 2022) |
| *OcSOS1* | Na^+^/H^+^ reverse transporter | Pump Na^+^out of the cell or store it in vacuoles | *O. coarctata* | (Roy and Chakraborty, 2018) |
| *LbSOS1* | Na^+^/H^+^ reverse transporter | Pump Na^+^out of the cell or store it in vacuoles | *L. bicolor* | (Li et al., 2020) |
| *CdSOS1* | Na^+^/H^+^ reverse transporter | Pump Na^+^out of the cell or store it in vacuoles | *C. dactylon* | (Yong et al., 2022) |
| *AmSOS1* | Na^+^/H^+^ reverse transporter | Pump Na^+^out of the cell or store it in vacuoles | *A. marina* | (Tang et al., 2025) |
| *AmbHLH091* | AmbHLH091 | Positive regulation of *AmSOS1* expression | *A. marina* | (Tang et al., 2025) |
| *AmNAC035* | AmNAC035 | Interacting with AmbHLH091 | *A. marina* | (Tang et al., 2025) |
| *OcHKT1;5* | High affinity potassium transporter | Pump Na^+^ into cells | *O. coarctata* | (Rajakani et al., 2019) |
| *LbHKT1*;*1* | High affinity potassium transporter | Pump Na^+^ into cells | *L. bicolor* | (Zhu et al., 2025b) |
| *LbNKCC* | Na^+^: K^+^: Cl^-^ cotransporter | Pump Na^+^out of the cell | *L. bicolor* | (Yang et al., 2012) |
| *LbNSCC* | non-selective cationic channel | Na^+^ is transported into the cell while K^+^ is transported out of the cell | *L. bicolor* | (Yuan et al., 2016a) |
| *LbCNGC* | cyclic nucleotide-gated cation channel | Pump Na^+^ into cells | *L. bicolor* | (Yuan et al., 2016a) |

**Supplementary Table 4 Comparison of the three major hypotheses for salt secretion by salt glands**

| Hypothesis / Characteristic | Core driving force | Gland type applicability | Ion selectivity | Key supporting evidence and references |
| --- | --- | --- | --- | --- |
| Osmotic mechanism hypothesis | Hydrostatic pressure (driven by osmosis) | Unicellular and bicellular salt glands | Non-selective | In *O. coarctata*, the unicellular salt glands on the lower leaf epidermis secrete Na^+^ by collapsing and rupturing (Sengupta and Majumder, 2009); the bicellular glands regulate Na^+^ transport by switching the morphology of their basal cells in response to osmotic pressure (Semenova et al., 2010). |
| Reverse pinocytosis hypothesis | Vesicle trafficking | Bicellular and multicellular salt glands | Selective | In bicellular salt glands, the vesicle-associated membrane protein SaVAMP plays a key role in salt secretion (Liu, 2011); in the multicellular salt glands of *L. bicolor*, the vesicular transport inhibitor BFA can inhibit salt secretion (Lu et al., 2020), and multiple vesicle transport-related proteins have been identified in their secreted proteome (Lu et al., 2022). Vesicular transport depends on membrane-localized ion transporters for ion loading and unloading, which confers ion selectivity (Gao et al., 2017). |
| Animal-like fluid transport hypothesis | Ion transporters and pumps | Unicellular, bicellular, and multicellular salt glands | Selective | OcNHX1 and OcHKT1;5 are expressed in unicellular salt glands (Rajakani et al., 2019); unicellular and bicellular salt glands possess a partitioning membrane system enriched with mitochondria and ion transporters (Koteyeva et al., 2022); the multicellular salt glands not only exhibit high expression of the H⁺-ATPase LbPMA (Ding et al., 2010), but also mediate salt secretion through channels such as LbNSCC and LbCNGC (Yuan et al., 2016a; Yuan et al., 2016b); this secretory function is inhibited by H⁺-ATPase inhibitors, Na⁺/K⁺-ATPase inhibitors, and NKCC inhibitors (Dschida et al., 1992; Balsamo et al., 1995; Ma et al., 2011; Yang et al., 2012). |
